# A Diagnostic Algorithm using Multi-parametric MRI to Differentiate Benign from Malignant Myometrial Tumors: Machine-Learning Method

**DOI:** 10.1038/s41598-020-64285-w

**Published:** 2020-05-04

**Authors:** Mahrooz Malek, Elnaz Tabibian, Milad Rahimi Dehgolan, Maryam Rahmani, Setareh Akhavan, Shahrzad Sheikh Hasani, Fatemeh Nili, Hassan Hashemi

**Affiliations:** 1 Advanced Diagnostic and Interventional Radiology Research Center (ADIR), Radiology Department, Imam Khomeini Hospital Complex (IKHC), Tehran University of Medical Sciences (TUMS), Tehran, No. 1419733141 Iran; 2 Faculty of Electrical Engineering, K.N. Toosi University of Technology, Tehran, No. 1631714191 Iran; 3 Gynecology Oncology Department, Imam Khomeini Hospital Complex (IKHC), Tehran University of Medical Sciences (TUMS), Tehran, No. 1419733141 Iran; 4 Pathology Department, Imam Khomeini Hospital Complex (IKHC), Tehran University of Medical Sciences (TUMS), Tehran, No. 1419733141 Iran

**Keywords:** Machine learning, Cancer imaging, Endometrial cancer

## Abstract

This study aimed to develop a diagnostic algorithm for preoperative differentiating uterine sarcoma from leiomyoma through a supervised machine-learning method using multi-parametric MRI. A total of 65 participants with 105 myometrial tumors were included: 84 benign and 21 malignant lesions (belonged to 51 and 14 patients, respectively; based on their postoperative tissue diagnosis). Multi-parametric MRI including T1-, T2-, and diffusion-weighted (DW) sequences with ADC-map, contrast-enhanced images, as well as MR spectroscopy (MRS), was performed for each lesion. Thirteen singular MRI features were extracted from the mentioned sequences. Various combination sets of selective features were fed into a machine classifier (coarse decision-tree) to predict malignant or benign tumors. The accuracy metrics of either singular or combinational models were assessed. Eventually, two diagnostic algorithms, a simple decision-tree and a complex one were proposed using the most accurate models. Our final simple decision-tree obtained accuracy = 96.2%, sensitivity = 100% and specificity = 95%; while the complex tree yielded accuracy, sensitivity and specificity of 100%. To summarise, the complex diagnostic algorithm, compared to the simple one, can differentiate tumors with equal sensitivity, but a higher specificity and accuracy. However, it needs some further time-consuming modalities and difficult imaging calculations. Trading-off costs and benefits in appropriate situations must be determinative.

## Introduction

There are many conditions in medicine that decision making plays a crucial role in the differentiation of binary diagnoses such as preoperative discrimination of benign from malignant uterine tumors. Physicians are not usually able to pool multiple parameters affecting the diagnosis, while “machine-learning” techniques can process such amounts of data to help physicians making their decisions more accurately. Simple “decision-tree”, as the most popular technique has the advantage to provide human-readable results with acceptable reliability^1,2^. This study aimed to develop some diagnostic models for preoperative differentiation of uterine sarcoma from benign leiomyoma using a supervised machine-learning method (i.e. machine classifier) based on “multi-parametric” magnetic resonance imaging (MRI).

“Leiomyoma” is the most common uterine tumor, affecting women of reproductive age, while different types of uterine sarcomas are rare, accounting for only 10% of uterine malignancies. It is associated with even a poorer prognosis than uterine adenocarcinoma^3–12^. Leiomyosarcoma causes about 26% of all deaths attributed to uterine malignancies^9^. Treatment of sarcoma is widely different from a benign uterine tumor. Leiomyoma mostly undergoes uterine-conservative management, while sarcoma needs hysterectomy with or without chemotherapy regimen. Among the different types of uterine sarcomas, “leiomyosarcoma” has several similarities with leiomyoma. Although some preoperative findings are beneficial for differentiation, there is a remarkable overlap in their characteristics. Currently, there are no reliable preoperative diagnostic criteria available. Actually, the ultimate diagnosis can be achieved only after the surgery, based on the histopathological examination^6,13–20^.

Previous studies have shown that MRI could be helpful in the differentiation of leiomyosarcoma from leiomyoma^3,21^. However, some of the degenerated and cellular leiomyoma might mimic sarcomas in the hemorrhage cystic degeneration and necrosis^22–30^. Therefore the “conventional” MRI sequences are not capable of making the ultimate diagnosis^3–5^. In this study, we have investigated whether the singular parameters (=features) extracted from lesions using multi-parametric MRI was significantly different between benign and malignant uterine tumors. Besides, the final goal of our study was to develop two groups of diagnostic algorithms (simple versus complex models) for preoperative discrimination of leiomyoma from leiomyosarcoma using machine classifiers made-up of various combination-sets of singular MRI features. The utilised imaging sequences for developing “simple” decision-tree were some feasible techniques such as diffusion-, T1- and T2-weighted images. On the other hand, the “complex” model was made-up of more advanced techniques including the aforementioned conventional sequences, plus quantitative T2, contrast-enhanced (CE) images, MR spectroscopy (MRS), and apparent diffusion coefficient (ADC) map. Another goal of this investigation was to evaluate the predictive value of these two models, comparing to that of our previous model^31^, based on dynamic contrast-enhanced (DCE-MRI).

## Results

### Characteristics of Two Types of Tumors

Characteristics of all included lesions, 84 benign and 21 malignant-tumors (belonged to 51 and 14 patients, respectively; based on their postoperative tissue diagnosis), were depicted in Table 1. The mean age of patients in malignant and benign groups did not differ significantly (39.5 versus 42.8 years, respectively; P value = 0.252). About 62% of this population were premenopausal women in both groups. Also, there was no significant difference between the mean lesion size of malignant and benign groups (79.5 versus 68.2 mm, respectively; P value = 0.70).

**Table 1:** Patients’ characteristics.

	No. of patients (%)	No. of lesions (%)	Premenopausal Proportion (%)	Age [year] mean ± SD (min - max)	Lesion size [mm] mean ± SD (min - max)
Benign	51 (78.5%)	84 (80%)	31:51 (62.0%)	42.8 ± 13.3 (21–66)	68.2 ± 41.8 (8–219)
Malignant	14 (21.5%)	21 (20%)	9:14 (62.5%)	39.5 ± 11.2 (18–68)	79.5 ± 49.5 (20–192)
Total	65	105	40:65 (62.3%)	42.1 ± 11.7 (18–68)	70.5 ± 45.1 (8–219)
p-value			0.995^**+**^	0.25^**++**^	0.70^**++**^

^+^Chi-square test; ^++^Two independent samples t-test; **SD**: Standard Deviation; **No**.: Number.

Table 2 has provided a comparison of all qualitative parameters between malignant and benign groups. All these categorical variables, except for the presence of hyper-signal areas on T1 (p value = 0.20), revealed a significant difference between the two groups. Table 3 has demonstrated a similar comparison of the quantitative parameters. All of them were significantly different between the two groups (p < 0.001). Also, the accuracy metrics for both quantitative (continuous) and qualitative (categorical) parameters have been presented in Table 4.

**Table 2:** Comparison of qualitative variables between malignant and benign groups.

Variable	Status	Benign (%)	Malignant (%)	Total (%)	p-value
Predominant high signal on T2*	No	59 (70.2%)	0 (0%)	59	0.001^**+**^
	Yes	25 (29.8%)	21 (100%)	46	
	Total	84 (0 missing)	21 (0 missing)	105	
Hyper signal areas on T1	No	78 (94%)	18 (85.7%)	96	0.20^**+**^
	Yes	5 (6%)	3 (14.3%)	8	
	Total	83 (1 missing)	21 (0 missing)	104	
Central Necrosis*	No	80 (95.2%)	11 (52.4%)	91	0.001^**+**^
	Yes	4 (4.8%)	10 (47.6%)	14	
	Total	84 (0 missing)	21 (0 missing)	105	
Restriction*	Negative	76 (95%)	0 (0%)	76	0.001^**+**^
	Positive	4 (5%)	21 (100%)	25	
	Total	80 (4 missing)	21 (0 missing)	101	
MRS Choline peak*	Negative	45 (92%)	4 (30%)	49	0.001^**+**^
	Positive	4 (8%)	9 (70%)	13	
	Total	49 (35 missing)	13 (8 missing)	62	
MRS Lipid peak*	Negative	48 (96%)	5 (39%)	53	0.001^**+**^
	Positive	2 (4%)	8 (61%)	10	
	Total	50 (34 missing)	13 (8 missing)	63	

^+^Chi-square test; *indicates a variable that led to a p < 0.001.

**Table 3:** Comparison of quantitative variables between benign and malignant groups.

Variable	Status	No. of patients	Mean ± SD	p-value	No. of Missing	Min _ Max
T2 Map^*^	B	68	66.78 ± 10.94	0.001^+^	17	47 _ 105
	M	20	93.15 ± 7.14			
T2 Scaled Ratio^*^	B	84	0.19 ± 0.18	0.0001^+^	1	−0.18 _ 1.01
	M	20	0.66 ± 0.21			
Tumor/Myometrium Ratio on T2^*^	B	82	−0.02 ± 0.61	0.0001^+^	4	−0.99 _ 2.19
	M	19	1.12 ± 0.55			
Tumor/Psoas Ratio on T2^*^	B	84	0.85 ± 1.02	0.0001^+^	0	−0.61 _ 5.48
	M	21	3.19 ± 1.23			
Tumor/Myometrium Ratio on CE^*^	B	82	0.00 ± 0.39	0.0001^+^	4	−0.75 _ 1.49
	M	19	0.61 ± 0.33			
Tumor/Psoas Ratio on CE ^*^	B	84	1.00 ± 0.44	0.0001^+^	0	−0.14 _ 2.97
	M	21	1.68 ± 0.58			
ADC mean*	B	80	1.426 ± 0.233	0.0001^+^	4	0.57 _ 2.37
	M	21	0.877 ± 0.384			

^+^Two independent samples t-test; *indicates a variable that led to a p<0.001; **B**: benign; **M**: malignant; **Min**: Minimum; **Max**: Maximum; **SD**: standard deviation; **No**.: number; **CE**: Contrast Enhanced images.

**Table 4:** Accuracy metrics for all singular features in distinguishing malignant from benign tumors.

Variable	Overall Accuracy (%)	AUC	Sen (%)	Spe (%)	NPV (%)	PPV (%)	BER (%)
Singular features							
Predominant high signal on T2	76.2	0.83	100	70	100	45	15
Hyper signal areas on T1	77.9	0.54	14	94	81	37	46
Tumor/Psoas Ratio on CE	81.9	0.77	24	96	84	63	40
Tumor/Myometrium Ratio on T2	82.9	0.81	43	93	87	60	32
Tumor/Psoas Ratio on T2	83.8	0.87	67	88	91	58	22.5
T2 Scaled Ratio	83.8	0.91	57	90	89	60	26.5
Central Necrosis	85.7	0.65	48	95	88	71	28.5
Tumor/Myometrium Ratio on CE	85.7	0.87	52	94	89	69	27
MRS Choline Peak	87.3	0.70	69	92	92	69	19.5
MRS Lipid Peak	88.9	0.69	62	96	91	80	21
Mean ADC	89.5	0.82	76	93	94	73	15.5
T2 Map	92.0	0.87	90	93	97	78	8.5
Restriction	96.2	0.96	100	95	100	84	2.5

The best two values in each column are indicated in underlined format. **AUC:** Area Under receiver operating Characteristics; **Sen:** sensitivity; **Spe:** specificity; **NPV**: Negative Predictive Value; **PPV:** Positive Predictive Value; **BER:** Balanced Error Rate; **CE:** Contrast Enhanced images.

### Discrimination Based on Singular Parameters

As we can see in Table 4, singular features are sorted by ascending rank of overall accuracy. DWI-Restriction, followed by T2-map and Mean-ADC, obtained the highest accuracy-values (96.2%, 92.0%, and 89.5%, respectively). The cut-off value for T2-map and mean-ADC were 79.5 and 1.06 × 10^−3^ mm^2^/s, respectively. The minimum balanced error-rate (BER) was recorded for Restriction (BER = 2.5%) and T2-map (8.5%). Only two singular features obtained a “sensitivity” of 100%; “Restriction, and predominant-T2-signal”. It means that by applying either of these features, none of the malignancies would have been misclassified into the benign group. However, the specificity was far significantly different between these two features (95% for Restriction versus 70% for T2-signal). The best specificity-value was calculated for the lipid-peak in MRS (96%), and TPCE-ratio (96%), followed by CN (95%). Furthermore, the best positive predictive value (PPV) was detected for Restriction (84%), and lipid-peak (80%).

### Discrimination Based on Combinational Models

Table 5 has shown the accuracy metrics for combinational trained models in two categories of simple and complex. In each category, by omitting less important parameters (those with the least accuracy, previously described in Table 4), we achieved “smaller models” without remarkable loss in accuracy. It is important to point out that a larger model is usually at a greater risk of over-fitting. In fact, the differentiation performance of a complex model on real population would be certainly much lower than that of the training sample.

**Table 5:** Accuracy metrics for combinational models in distinguishing malignant from benign tumors.

Model No.	Model Category	No. of features	AUC	Sen (%)	Spe (%)	NPV (%)	PPV (%)	Accuracy (%)
	**Simple Models**							
A: _Original_ [1]	Restriction + CN + T2 + T1	4	0.96	100	95	100	84	96.2
2	Original - T2	3	0.96	100	95	100	84	96.2 → ^¤^
3	Original - T1	3	0.96	100	95	100	84	96.2→
4	Original - CN	3	0.95	100	94	100	81	95.2↓
5	Original - Restriction	3	0.89	48	99	88	91	88.6↓
*6	Original - (T2 + T1)	2	0.96	100	95	100	84	96.2 →
→The best Model = [Restriction + CN]								
	**Complex Models**							
B: _Original_ [7]	Qualitative Features:Restriction+ CN + Lipid peak +Choline peak+T2 + T1	6	0.96	95	98	99	91	97.1
8	Original - (T2 signal + T1 signal)	4	0.96	95	98	99	91	97.1→
9	Original - (Lipid peak + Choline peak)	4	0.96	100	95	100	84	96.2↓
*10	Original - (Choline peak + T2 signal + T1 signal)	3	0.97	95	99	99	95	98.1↑
11	Original - (Lipid peak + T2 signal + T1 signal)	3	0.96	95	98	99	91	97.1→
12	Original - (Lipid peak + Choline peak + T2 signal + T1 signal)	2	0.96	100	95	100	84	96.2↓
13	Original - (CN + Restriction + T2 signal + T1 signal)	2	0.69	46	92	87	60	82.5↓
14	Original - (CN + T2 signal + T1 signal)	3	0.96	100	95	100	84	96.2↓
15	Original - (CN + Choline peak + T2 signal + T1 signal)	2	0.96	100	95	100	84	96.2↓
16	Original - (Restriction + Choline peak + T2 signal + T1 signal)	2	0.84	62	93	91	68	86.7↓
→The best Model = [Restriction + CN + Lipid peak]								
C: _Original_ [17]	Quantitative Features:T2 map + Mean ADC + T2 scaled + 4 TM/TP ratios	7	0.85	76	94	94	76	90.5
18	Original - (T2 map + mean ADC + T2 scaled)	4	0.86	81	87	95	61	85.7↓
19	Original - (TMCE Ratio + TP Ratio + TM Ratio + TPCE Ratio)	3	0.92	86	94	96	78	92.4↑
20	Original - (TP Ratio + TM Ratio + TPCE Ratio)	4	0.92	86	95	96	82	93.3↑
21	Original - (TMCE Ratio + TM Ratio + TPCE Ratio)	4	0.89	86	93	96	75	91.4↑
22	Original - (TMCE Ratio + TP Ratio + TPCE Ratio)	4	0.92	81	94	95	77	91.4↑
23	Original - (TMCE Ratio + TM Ratio + TP Ratio)	4	0.88	76	95	94	80	91.4↑
*24	Original - (T2 scaled + TP Ratio + TM Ratio + TPCE Ratio)	3	0.89	81	98	95	89	94.3↑
25	Original - (Mean ADC + TP Ratio + TM Ratio + TPCE Ratio)	3	0.89	81	95	95	81	92.4↑
26	Original - (T2 map + TP Ratio + TM Ratio + TPCE Ratio)	3	0.94	90	94	98	79	93.3↑
27	Original - (TMCE Ratio + T2 scaled + TP Ratio + TM Ratio + TPCE Ratio)	2	0.91	76	95	94	80	91.4↑
28	Original - (Mean ADC + T2 scaled + TP Ratio + TM Ratio + TPCE Ratio)	2	0.85	76	96	94	84	92.4↑
29	Original - (T2 map + T2 scaled + TP Ratio + TM Ratio + TPCE Ratio)	2	0.86	86	95	96	82	93.3↑
→The best Model = [T2 map + Mean ADC + TMCE Ratio]								
D: _Original_ [30]	Total: Restriction+ T2map+ mean ADC + TMCE Ratio+ CN + Lipid peak	6	0.98	95	100	99	100	99.0
31	Original - (Lipid peak)	5	0.98	95	100	99	100	99.0→
32	Original - (CN + Lipid peak)	4	0.98	95	100	99	100	99.0→
33	Original - (TMCE Ratio + Lipid peak)	4	0.98	95	100	99	100	99.0→
*34	Original - (Mean ADC + Lipid peak)	4	1	100	100	100	100	100↑
35	Original - (T2map + Lipid peak)	4	0.96	95	96	99	87	96.2↓
36	Original - (Restriction + Lipid peak)	4	0.87	81	96	95	85	93.3↓
37	Mean ADC + Lipid peak	2	0.93	62	100	91	100	92.1↓
38	Mean ADC + (TM Ratio or TMCE Ratio)	2	0.86	86	95	96	82	93.3↓
39	Restriction + Mean ADC + T2 signal	2	0.95	95	96	99	87	96.2↓
→The best Model = [Restriction + T2 map + TMCE Ratio + CN]								

¤Arrows indicate the direction of changes. *indicates the best models in each category. **No**.:number; **AUC:** Area Under receiver operating Characteristics; **Sen:** sensitivity; **Spe:** specificity; **NPV:** Negative Predictive Value; **PPV:** Positive Predictive Value; **CN:** Central Necrosis; **TP Ratio:** Tumor/Psoas Ratio on T2; **TM Ratio:** Tumor/Myometrium Ratio on T2; **TPCE Ratio:** Tumor/Psoas Ratio on Contrast Enhanced images; **TMCE Ratio:** Tumor/Myometrium Ratio on Contrast Enhanced images.

### Training Simple Models

**Figure 1:**
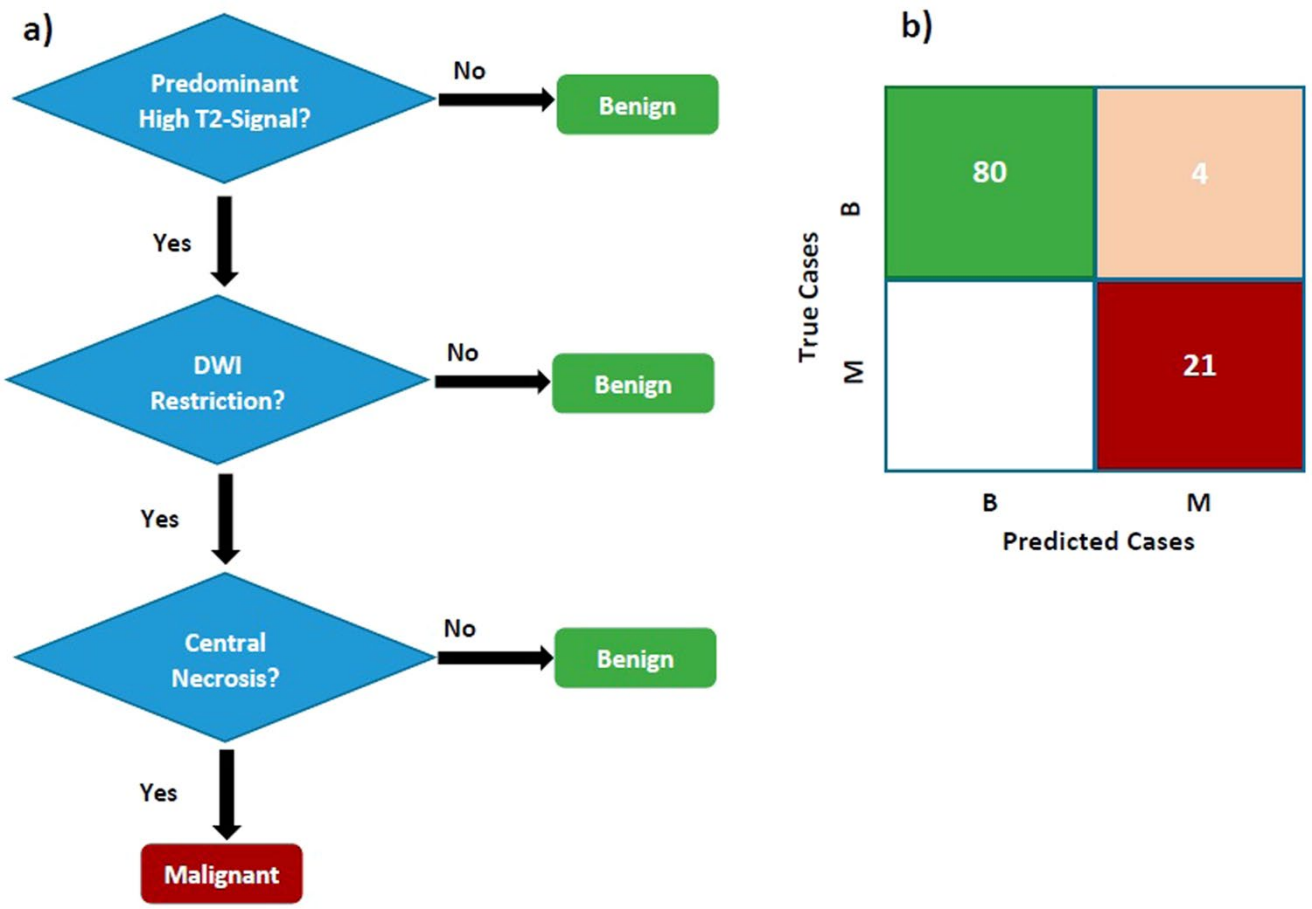
(**a**) Simple decision-tree using 3 parameters of predominant T2-signal, Restriction and Central Necrosis. (**b)** Confusion-matrix for the number of lesions on true and false predicted classes. B: Benign; M: Malignant.

Selected features in our simple models were the categorical ones including Restriction in diffusion-weighted images (DWI), predominant high-signal on T2, central-necrosis (CN), and the presence of high-signal areas on T1. Among them, the model (training#6 or t#6) that combined two parameters of DWI-Restriction, and CN was selected as the best one [Table 5, part A]. However, since our goal was using a multi-parametric approach, T2-sequence, due to its wide availability, was added to the final algorithm [Fig. 1], without any decline in the accuracy (96.2%). Clearly, the machine placed the parameters with the highest sensitivity at the top; and those with the maximum amount of specificity at the lower nodes of the tree. Therefore, the predominant high-signal on T2 was placed at the beginning node, as the first question. If the answer to the latter was negative for a lesion, then it could be certainly classified as benign lesion. However in another branch, at the expense of misclassifying 30% of benign cases (specificity of 70% for T2), all malignant-lesions would have been correctly categorised. Here, the next two nodes, i.e. DWI-Restriction and CN, would do their best to strongly detect the remaining benign cases [Fig. 1]. Therefore, the final branch would contain all malignant cases (sensitivity = 100%; but PPV = 84%), as well as the remaining benign cases (specificity = 95%; and NPV = 100%).

### Training Complex Models (qualitative parameters)

In order to determine the best complex model, data were divided into two sub-classes of “qualitative” and “quantitative” sections. We initially combined all our six quantitative parameters into a single model (t#7) which showed an accuracy of 97.1% and AUC of 0.96. Then, by removing T1 and T2-parameters (t#8), we achieved a smaller model with equal accuracy. On the other hand, the omission of two MRS-peaks led to a lower accuracy; i.e. t#9 (accuracy = 96.2%). Also, a similar decline was seen when CN and choline-peak would have been deleted (t#15). The next set was combining two MRS-peaks, with and without the aforementioned simple features (CN and Restriction). MRS-peaks alone could only obtain an accuracy of 82.5% (t#13). By comparing model#10 and #16, we could realise that DWI is highly valuable and essential. Adding DWI significantly improved the model accuracy to 96.2% (t#14). Finally, model#10 with an accuracy of 98.1% was elicited as the best one in the qualitative section [Table 5, part B]. The performance of this model was slightly better than the best simple one (t#6; 96.2%).

### Training Complex Models (quantitative parameters)

In the “quantitative” section, the predictive performance of the original model comprised of all 7 features was about 90.5% (t#17). Combination of other features, i.e. four comparative ratios including Tumor-Myometrium contrast ratio on CE (TMCE-Ratio), Tumor-Psoas contrast ratio on CE (TPCE-Ratio), Tumor-Myometrium contrast ratio on T2 (TM-Ratio), and Tumor-Psoas contrast ratio on T2 (TP-Ratio) showed a poor accuracy and sensitivity (t#18 with 85.7% and 81%, respectively). In fact, this classifier would categorise 19% of malignant-lesions into the benign group. By removing these four features from the original model, better accuracy of 92.4% was yielded (t#19). All other possible combination sets were trained. However, “only” the most important ones have been presented in Table 5. Eventually, the t#24 which was consisted of three features of T2-map, mean-ADC, and TMCE-ratio, with an overall accuracy of 94.3% was elicited as the best one [Table 5, part C]. It is noteworthy to mention that the “minimum” number of 2 and 4 features were considered to train the models in simple and complex sections, respectively.

### Eliciting the Final Complex Model

In the last part of Table 5, we combined the best “qualitative and quantitative” parameters into a final category. The original model in the last section (t#30), resulted in an accuracy of 99.0%. Since lipid-peak did not add any further prediction-value (t#31), it was no longer wise to perform MRS, while CN could play a similar role. Among the other five parameters, further deletion of mean-ADC provided even higher accuracy (t#34). The latter was the most valuable model comprised of two quantitative features (T2-map and TMCE-Ratio), and two qualitative ones (Restriction and CN), showing 100% of all accuracy indices [Table 5, part D]. Again, the prediction value of Restriction and T2-map as the best valuable features was examined by removing them (t#35,36).

**Figure 2:**
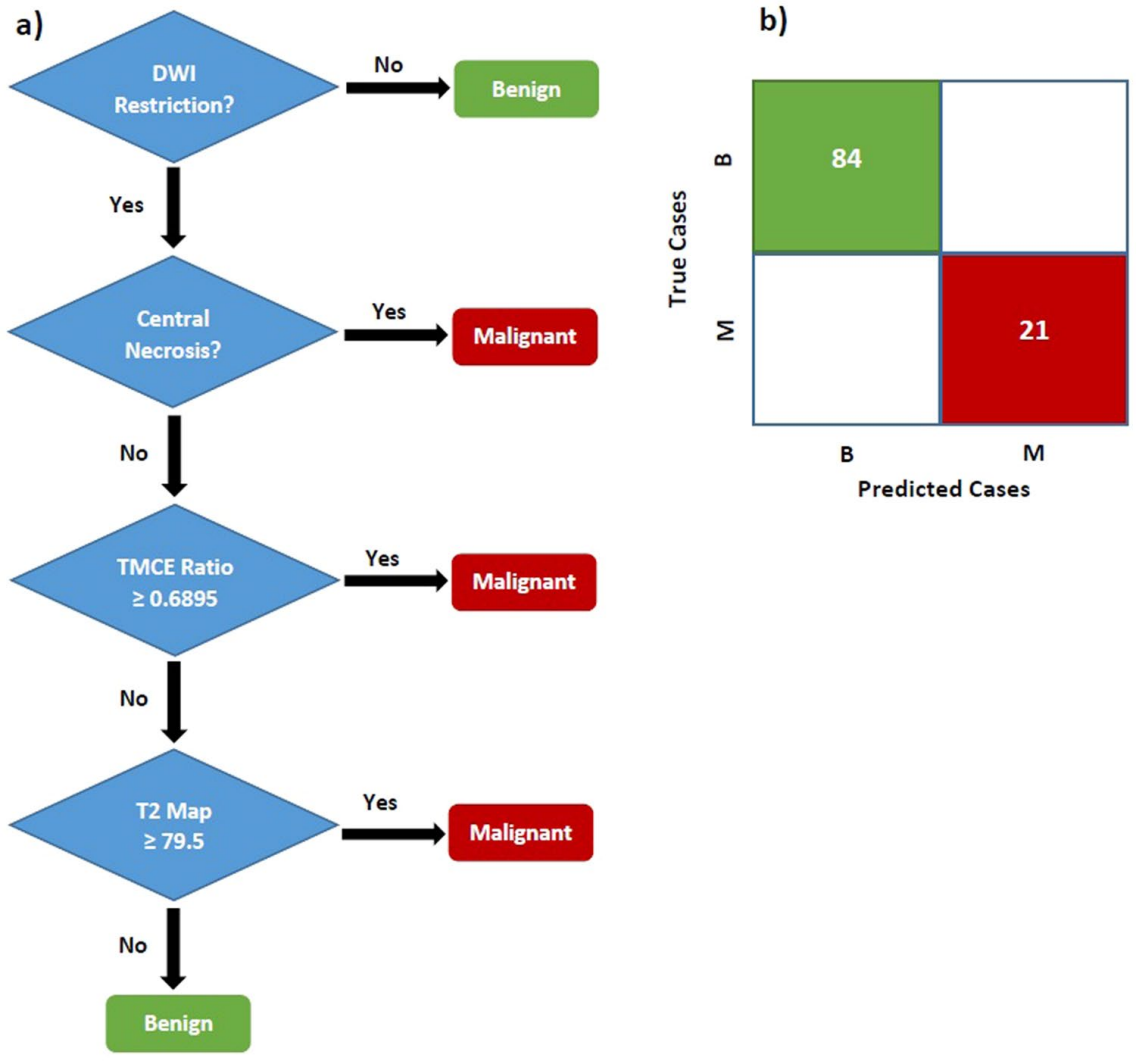
(**a**) Complex decision-tree using 4 parameters Restriction, Central Necrosis, T2-map, and TMCE-Ratio. (**b)** Confusion-matrix for the number of lesions on true and false predicted classes. B: Benign; M: Malignant.

Moreover, according to the suggestion of earlier studies, we also trained some smaller models (t#37 to 39). A model consisted of lipid-peak in MRS and mean-ADC value revealed a specificity of 100% and accuracy of 92.1%. Despite such a high specificity, this model had still a lower accuracy than the best complex model. Another possible combination proposed by some studies was the mean-ADC value, added to quantitative T2-parameters (e.g. TMCE-ratio). This classifier (t#38) obtained an accuracy of 93.3%. Another model was Restriction combined with mean-ADC value and T2-signal-intensity that showed an accuracy of 96.2% (t#39). Eventually, since none of these models provided higher accuracy, we elicited model#34 as the best final complex model. This model was able to correctly predict all 105 lesions of the present dataset. The corresponding decision-tree has been depicted in Fig. 2 > . According to the algorithm, we can find out that if a lesion shows restriction on DW image, it could be certainly labeled as malignant tumor unless the next three questions (presence of CN, TMCE ratio ≥0.6895 and T2-map ≥79.5) would obtain negative (No) answers. Theoretically, this algorithm would detect benign and malignant myometrial lesions with NPV, PPV, Sen and Spe of 100% [Fig. 2].

## Discussion

Prior studies have recommended that MRI could be a reliable method to discriminate between benign and malignant uterine masses. This study has used a supervised machine-learning model based on multi-parametric MRI including conventional-sequences, with and without advanced techniques. A general assessment of Restriction based on DWI and ADC-map was the most valuable feature in our data which complies with the existing literature^30,34^. Sato et al. found a specificity and accuracy of 94% with 100% of sensitivity. They concluded that all lesions exhibiting low signal-intensity on DWI were benign-tumors; whereas in cases of a high or intermediate-signal, mean-ADC should be measured. The diagnosis would be leiomyosarcoma or atypical leiomyoma if the value turned out to be lower than 1.1 × 10^−3^ mm^2^/s^30^. Their reported accuracy was close to the corresponding model in our research (t#38; accuracy = 95.2%, sensitivity and specificity = 95%). Another study by Li et al. indicated that the mean-ADC value was significantly different between malignant and benign-lesions, with an accuracy of 100%^34^. The mean-ADC in our research, correctly discriminate 89.5% of lesions with the cut-off point = 1.06×10^−3^ mm^2^/s; ranking as the third-highest accuracy [Table 4].

The present study also confirmed that a positive peak in MRS could highly be suggestive for the malignancy. This finding was in line with previous studies^35,36^. However, our sensitivity and specificity were much lower than those of Takeuchi (100% and 96%, respectively)^36^. These variations could be attributed to the differences in the nature of included masses, either endometrial or myometrial lesions. Generally, positive lipid-peak was a more accurate indicator of malignancy, in comparison to choline-peak [Table 4]. From a molecular viewpoint, positive lipid-peak in MRS was parallel to the presence of CN in T2-images of leiomyosarcoma. As expected, these two features showed a similar high specificity-value, as opposed to a poor sensitivity [Table 4]. We also investigated the added benefit of MRS to mean-ADC in a combinational-model (t#37), resulting in an accuracy of 92.1%. However, we did not observe any additional benefit when utilizing either lipid-peak or mean-ADC (model#34) in our final model, thereby eliminating these variables from the algorithm. Rather, CN and Restriction were more “feasible” factors. Also, our results of quantitative T2-parameters were relatively consistent with earlier findings^37,38^. The current data on T2-map, as the second most important feature, revealed accuracy and error-rate values of 92.0% and 8.5%, respectively [Table 4]. In two other studies^22,29^, the diagnostic benefit of combining T2-weighted and DW-images were investigated. One study achieved an accuracy of 92.4% and PPV of 92%, by combining T2, DWI, and mean-ADC value^29^. A similar model (t#39) in our study achieved 96.2% accuracy and negative predictive value (NPV) = 99%. Another research proved that using tumor/myometrium signal ratio on T2 (similar to TM-Ratio in our study)^22^, combined with mean-ADC, could classify tumors with an accuracy and specificity of 100% which is much higher than our results (t#38).

An article of the present authors in 2019, evaluated the DCE-MRI performance to differentiate malignant and benign uterus tumors^31^. The findings revealed an accuracy of 92%, the sensitivity of 100%, and specificity of 90%. However, it should be pointed out that DCE-MRI is not a routine technique. Additionally, that method of statistical analysis was an intricate method known as “ensemble of bagged-trees”, without any human-readable result. In the current study, we have tried to address this limitation by using simple decision-tree and dividing trained models into two simple and complex categories. Therefore, it might be slightly more severe than the actual population. However, in order to confirm the tissue diagnosis, this issue was deemed inevitable. Moreover, such as all similar studies, our sample size was small. Although the incidence of uterine sarcoma is very low, further validation of the current algorithms using larger samples is necessary.

In summary, this study discussed the application of multi-parametric MRI in the preoperative differentiation of benign from malignant myometrial tumors, using a supervised machine-learning method. We have also proposed two decision-trees. The best simple and complex decision-trees obtained accuracy percentages of 96.2 and 100, respectively. Both of them achieved better accuracy values in comparison to DCE-MRI with an associated accuracy of 92%^31^. The complex model differentiated tumors with higher accuracy and specificity than the simple one. However, the former needs more advanced calculations and a high level of patient’s cooperation, possibly making it a time-consuming method. Moreover, it should be kept in mind that the best overall score might not always indicate the best model. Therefore, the physician should wisely compromise between costs and benefits in appropriate clinical situations.

## Methods

### Study Design

After approving the protocol by our institutional (Tehran University of Medical Sciences) review board, a multi-parametric MRI was preoperatively performed for all participants with suspicious myometrial masses who were referred from gynecology-clinic during 2017. All of the recruited patients were candidates for myomectomy or hysterectomy (open surgery or laparoscopic). A written informed consent form was then administered for each patient. Eventually, 65 “women” who confirmed to have a total of 105 “lesions” were included [Table 1]. Some patients (not all of them) had more than one lesion; and theoretically different pathology results could be encountered. In order to avoid loss of data, we decided to include all lesions of any patient and increase the sample size, as well as enhancing the study power. However, there was no discrepancy in classifying multiple lesions of a patient. Actually in our dataset, no patient had both benign and malignant tumors simultaneously. The demographics and menopausal status of participants were collected. After surgery, the number and size of lesions, as well as the definitive diagnosis for each lesion, were determined according to the consensus of our pathologic department. All methods of this research were performed in accordance with the relevant medical ethics guidelines and regulations. The personal information of patients that could lead to the identification of a participant was not published and will remain confidential. Therefore, we received the confirmation of Tehran University of Medical Sciences (TUMS) Ethics Committee under number: IR.TUMS.IKHC.REC.1396.456.8.

### Imaging protocol

All imaging sequences were performed using a 3-T MR scanner. The conventional-sequences were T1, T2, and DWI with ADC-map. On the other hand, quantitative T2, CE-MRI, and MRS comprised the advanced modalities. Routine pelvic MRI protocol was performed prior to the acquisition of other sequences. Subjects were positioned supine on the MR-scanner table with the 4-channel phased-array coil placed over the pelvis. Moreover, CE-MRI was produced using an injection of gadolinium contrast medium (at 0.2 mmol/kg dose) about 120–180 seconds after the injection^32^. DWI was also acquired by using a single-shot echo-planar sequence in the axial plane with a section thickness of 4 mm, an intersection gap of 0.8 mm, and 280 mm field-of-view. DW images were acquired in three gradient directions. By referring T1- and T2-weighted images, as well as DWI, a technologist under the guidance of our senior radiologist (M.M) placed a single 2×2×2_cm3_ cubic spectroscopic volume-of-interest over the mass areas so that cystic or necrotic areas, large vessels, calcification, and hemorrhage were excluded as much as possible. Patients also underwent Proton MRS. However, according to the background noise, only about 60% of MRS data were approved for the final analysis by our physicist.

### Image analysis

As we described, 13 singular features were extracted and categorised in two parts: six qualitative and seven quantitative ones: Four qualitative features were used to develop the “simple” diagnostic models including

1. Presence of hyper-signal areas on T1;
2. Predominant signal on T2, expressed as high or low, compared to the outer myometrium;
3. Presence of well-defined CN on T2 image or contrast-enhanced (CE) T1 sequence; (The scattered necrosis was not accounted)
4. Visual assessment of restriction on DWI and ADC-map (=Restriction).

The qualitative evaluation of these four features was decided in a committee of three experienced radiologists (M.M; M.R; and H.H). These assessor physicians were blinded to the patient’s category.

The two other qualitative features were: (5) Lipid and (6) Choline-peaks observed in MRS. These MRS peaks, plus the following 7 quantitative features, were combined to make a “complex” diagnostic model.

(7) Mean-ADC value was automatically generated on a pixel-by-pixel basis using Formula-1, where b_0_ and b_1_ represent lower and upper b-values and were set to 0 and 1000 _sec/mm_^2^, respectively. S_0_ and S_1_ were the corresponding signal intensities for these b-values. To calculate the mean “ADC” value of lesion, we determined the ROI that encompassed the solid part of the tumor. Beside this calculation, the general signal intensity of DW-images for a b-value of 1000 _s/mm_^2^ and the corresponding signal on ADC-map was visually assessed. When the lesion had high signal on DW-images and low on ADC, it was considered as a restricted lesion.

1 $${\rm{Diffusion}}\,{\rm{coefficiency}}=\frac{-(\mathrm{ln}({S}_{1})-\,\mathrm{ln}({S}_{0}))}{({b}_{1}-{b}_{0})}$$

The other six quantitative parameters were extracted from T2-and CE-sequences as the following:

(8) T2-mapping (T2-map);

(9) T2-scaled ratio (T2-scaled);

(10) Tumor-Myometrium contrast ratio on T2 (TM-Ratio);

(11) Tumor-Psoas contrast ratio on T2 (TP-Ratio);

(12) Tumor-Myometrium contrast ratio on CE (TMCE-Ratio), and

(13) Tumor-Psoas contrast ratio on CE (TPCE-Ratio).

All quantitative metrics were calculated off-line using the PACS system by two independent experienced radiologists in gynecology-oncology imaging (M.R; and M.M). To calculate T2-map, a two-dimensional multi-echo (six TE-values from 10 to 61 ms) spin-echo sequence was utilised. In order to calculate T2-scaled using formula-2, a region of interest (ROI) was manually outlined which encompassed the entire tumor, while avoiding healthy tissue. T2-scaled ranged from 0 to 1 that 1 indicated the intensity of fat and 0 represented the intensity of rectus abdominis muscle.

2 $$T2\,Scaled\,Ratio=\frac{{\rm{Signal}}\,{\rm{intensity}}\,{\rm{of}}\,{{\rm{ROI}}}_{Mass}-{\rm{Signal}}\,{\rm{intensity}}\,{\rm{of}}\,{{\rm{ROI}}}_{{\rm{Rectus}}}}{{\rm{Signal}}\,{\rm{intensity}}\,{\rm{of}}\,{{\rm{ROI}}}_{Fat}-{\rm{Signal}}\,{\rm{intensity}}\,{\rm{of}}\,{{\rm{ROI}}}_{{\rm{Rectus}}}}$$

On T2-images, the largest possible ROI was placed over the mass, while cystic or necrotic areas, large vessels, calcification, and hemorrhage were avoided. Also, two ROIs that included the normal outer myometrium and psoas muscle was defined. According to these two ROIs TM- and TP-Ratios on T2 were calculated using Formula-3 and -4. Additionally, we calculated the TMCE- and TPCE-ratios on CE-images at the equilibrium phase in the same way.

3 $$\frac{{\rm{T}}{\rm{u}}{\rm{m}}{\rm{o}}{\rm{r}}}{{\rm{M}}{\rm{y}}{\rm{o}}{\rm{m}}{\rm{e}}{\rm{t}}{\rm{r}}{\rm{i}}{\rm{u}}{\rm{m}}}{\rm{S}}{\rm{i}}{\rm{g}}{\rm{n}}{\rm{a}}{\rm{l}}\,{\rm{R}}{\rm{a}}{\rm{t}}{\rm{i}}{\rm{o}}\,{\rm{o}}{\rm{n}}\,{\rm{T}}2\,{\rm{o}}{\rm{r}}\,{\rm{C}}{\rm{E}}=\frac{{\rm{S}}{\rm{i}}{\rm{g}}{\rm{n}}{\rm{a}}{\rm{l}}\,{\rm{i}}{\rm{n}}{\rm{t}}{\rm{e}}{\rm{n}}{\rm{s}}{\rm{i}}{\rm{t}}{\rm{y}}\,{\rm{o}}{\rm{f}}\,{{\rm{R}}{\rm{O}}{\rm{I}}}_{Mass}-{\rm{S}}{\rm{i}}{\rm{g}}{\rm{n}}{\rm{a}}{\rm{l}}\,{\rm{i}}{\rm{n}}{\rm{t}}{\rm{e}}{\rm{n}}{\rm{s}}{\rm{i}}{\rm{t}}{\rm{y}}\,{\rm{o}}{\rm{f}}\,{{\rm{R}}{\rm{O}}{\rm{I}}}_{{\rm{O}}{\rm{u}}{\rm{t}}{\rm{e}}{\rm{r}}{\rm{m}}{\rm{y}}{\rm{o}}{\rm{m}}{\rm{e}}{\rm{t}}{\rm{r}}{\rm{i}}{\rm{u}}{\rm{m}}}}{{\rm{S}}{\rm{i}}{\rm{g}}{\rm{n}}{\rm{a}}{\rm{l}}\,{\rm{i}}{\rm{n}}{\rm{t}}{\rm{e}}{\rm{n}}{\rm{s}}{\rm{i}}{\rm{t}}{\rm{y}}\,{\rm{o}}{\rm{f}}\,{{\rm{R}}{\rm{O}}{\rm{I}}}_{{\rm{O}}{\rm{u}}{\rm{t}}{\rm{e}}{\rm{r}}{\rm{m}}{\rm{y}}{\rm{o}}{\rm{m}}{\rm{e}}{\rm{t}}{\rm{r}}{\rm{i}}{\rm{u}}{\rm{m}}}}$$

4 $$\frac{{\rm{T}}{\rm{u}}{\rm{m}}{\rm{o}}{\rm{r}}}{{\rm{P}}{\rm{s}}{\rm{o}}{\rm{a}}{\rm{s}}}{\rm{S}}{\rm{i}}{\rm{g}}{\rm{n}}{\rm{a}}{\rm{l}}\,{\rm{R}}{\rm{a}}{\rm{t}}{\rm{i}}{\rm{o}}\,{\rm{o}}{\rm{n}}\,{\rm{T}}2\,{\rm{o}}{\rm{r}}\,{\rm{C}}{\rm{E}}\,=\frac{{\rm{S}}{\rm{i}}{\rm{g}}{\rm{n}}{\rm{a}}{\rm{l}}\,{\rm{i}}{\rm{n}}{\rm{t}}{\rm{e}}{\rm{n}}{\rm{s}}{\rm{i}}{\rm{t}}{\rm{y}}\,{\rm{o}}{\rm{f}}\,{{\rm{R}}{\rm{O}}{\rm{I}}}_{Mass}-{\rm{S}}{\rm{i}}{\rm{g}}{\rm{n}}{\rm{a}}{\rm{l}}\,{\rm{i}}{\rm{n}}{\rm{t}}{\rm{e}}{\rm{n}}{\rm{s}}{\rm{i}}{\rm{t}}{\rm{y}}\,{\rm{o}}{\rm{f}}\,{{\rm{R}}{\rm{O}}{\rm{I}}}_{{\rm{P}}{\rm{s}}{\rm{o}}{\rm{a}}{\rm{s}}}}{{\rm{S}}{\rm{i}}{\rm{g}}{\rm{n}}{\rm{a}}{\rm{l}}\,{\rm{i}}{\rm{n}}{\rm{t}}{\rm{e}}{\rm{n}}{\rm{s}}{\rm{i}}{\rm{t}}{\rm{y}}\,{\rm{o}}{\rm{f}}\,{{\rm{R}}{\rm{O}}{\rm{I}}}_{{\rm{P}}{\rm{s}}{\rm{o}}{\rm{a}}{\rm{s}}}}$$

Also, the visual detection of two apparent resonance peaks at 1.33ppm and 3.23ppm in MRS was considered positive for lipid and choline, respectively. Further explanation for the entire protocol and formulations can be found in our previous papers^32,33^.

### Statistical analysis, model validation and verification

The patients’ age, lesion size and menopausal status between the malignant and benign groups were compared using two independent samples t-test to assure that they were relatively matched. Normal distribution was checked using the Shapiro-Wilk test which resulted in a non-significant p-value for all variables. Therefore, chi-square and student’s t-test in SPSS software (IBM Corp., Armonk®), were utilised to compare the qualitative and quantitative parameters, respectively. A p-value of less than 0.05 was considered significant. Besides, we used machine classifier extension of MATLAB 2017a software (Mathworks, Natick®) to make some combinational-models (here, decision-tree), by which the machine categorises lesions. The mentioned qualitative and quantitative variables were step-by-step fed into a classifier. To evaluate the predictive performance of singular parameters, as well as the combinational-models, we generated Receiver-Operating-Characteristic (ROC) curves. The area under the curve (AUC) was then calculated. Additionally, by applying the optimal cut-off point in ROC curve, the accuracy metrics including sensitivity (Sen), specificity (Spe), overall accuracy, negative-predictive-value (NPV), positive-predictive-value (PPV), and balanced error-rate (=BER, based on Formula-5) were extracted for each feature.

BER = 1-Balanced Accuracy Rate (BAR) → BAR = (Sensitivity + Specificity)/2 (equation-5)

Evidently, the learned rules for associating the features to the proper label might be over-fitted for the current data-set. To avoid this problem, the k-fold cross-validation (CV) method was used (k = 10). In order to simplify, we have trained only coarse decision-tree models with the maximum number of 5 splits [as opposed to fine decision-trees with a maximum depth of 100 splits]. Feature-selection was done according to the evaluation of “scatter-plot”, “parallel-coordinates plot” and “confusion-matrix”. Eventually, the best “models” and their corresponding “decision-trees”, as the final algorithms, were selected.

**Figure 3:**
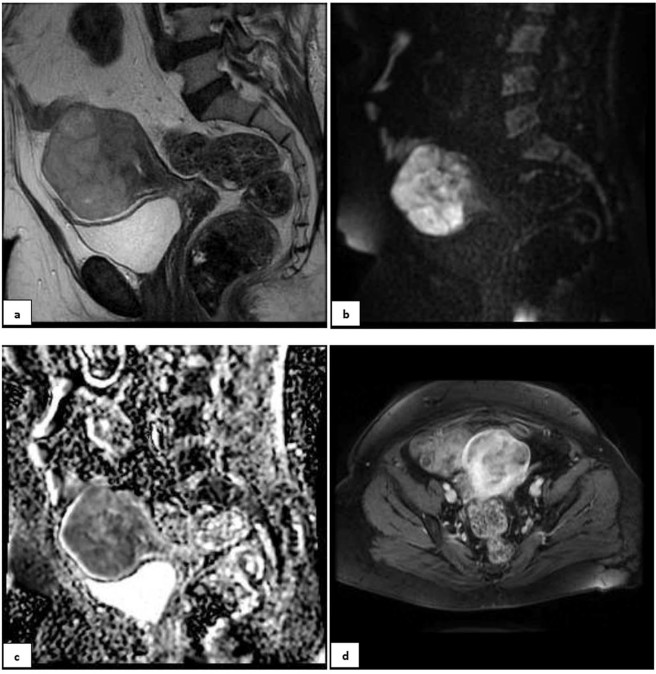
A 55-year-old postmenopausal patient with 3 months of abnormal uterine bleeding and a hypervascular myometrial mass on her ultrasound. (**a)** Sagital T2 MR-image detected a large predominantly hyper-signal lesion in anterofundal myometrium with extension to endometrial canal; Tumor-Myometrial Contrast (TM) Ratio = 1.98; T2-scaled Ratio = 1.01 and T2 map = 81. (**b)** Sagital-DW image and (**c)** ADC revealed restriction with mean ADC of 0.72 mm/s^2^. (**d)** Axial post contrast T1 image in equilibrium phase showed the mass has central necrosis and Tumor-Myometrium Contrast Enhanced (TMCE) Ratio = 1.1. If we put this sample data in either of simple or complex decision-tree, the pathology could be predicted as “malignant”. Eventually, the histo-pathological exam confirmed malignancy, a high grade sarcoma.

**Figure 4:**
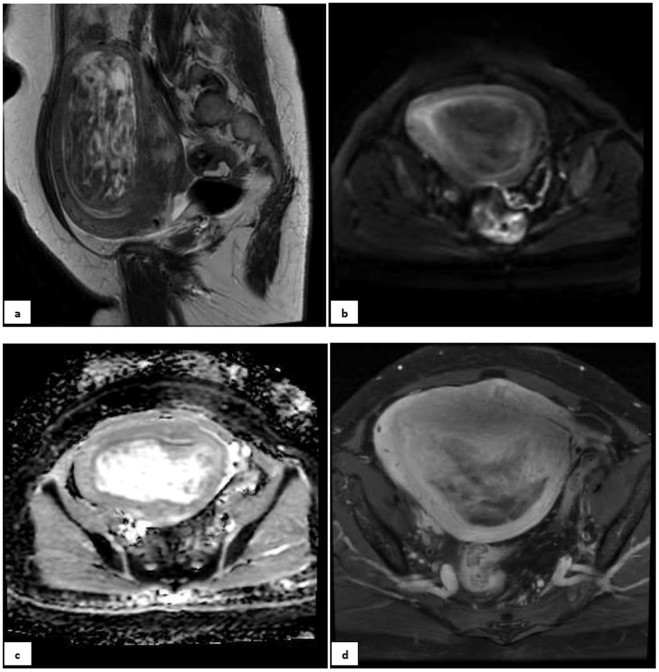
A 32-year-old nulliparous patient with 6 months of abnormal uterine bleeding and a heterogeneous myometrial mass on her ultrasound. (**a)** Sagital T2 MR-image detected a large predominantly hyper-signal lesion in posterofundal myometrium with anterior endometrial displacement; Tumor-Myometrial Contrast (TM) Ratio = 0.83; T2-scaled Ratio = 0.2 and T2 map = 42. (**b)** Axial DW image and (**c)** ADC revealed no evidence of restriction with mean ADC of 1.3 mm/s^2^. (**d)** Axial post-contrast T1 image in equilibrium phase showed the mass with mild enhancement significantly less than myometrium and without any central necrosis. Tumor-Myometrium Contrast Enhanced (TMCE) Ratio = −0.64. If we put this sample data in either of simple or complex decision-tree, the pathology could be predicted as “benign”. Eventually, the histo-pathological exam confirmed a benign tumor, degenerated leiomyoma.

For verification of the models, MR images of two sample patients, randomly selected, have been depicted in Figs. 3 and 4. If we enter the patients‘ data in either of simple or complex decision-tree, the predicted diagnosis could be compared against the gold-standard result.

## Data Availability

The datasets generated during and analysed during the current study are available from the corresponding author on reasonable request.
